# Effects of Glucagon-Like Peptide-1 Receptor Agonist Exendin-4 on the Reinstatement of Cocaine-Mediated Conditioned Place Preference in Mice

**DOI:** 10.3389/fnbeh.2021.769664

**Published:** 2022-01-05

**Authors:** Changliang Zhu, Lei Wang, Jiangwei Ding, Hailiang Li, Din Wan, Yangyang Sun, Baorui Guo, Zhenquan He, Xiaofan Ren, Shucai Jiang, Caibing Gao, Hua Guo, Tao Sun, Feng Wang

**Affiliations:** ^1^Department of Neurosurgery, General Hospital of Ningxia Medical University, Yinchuan, China; ^2^Ningxia Key Laboratory of Cerebrocranial Disease, Incubation Base of National Key Laboratory, Ningxia Medical University, Yinchuan, China; ^3^Department of Neurosurgery, The Second Affiliated Hospital of Nanchang University, Nanchang, China; ^4^Department of Neurosurgery, The First Affiliated Hospital of Zhejiang University School of Medicine, Hangzhou, China

**Keywords:** cocaine, reinstatement, exendin-4, pretreatment, nuclear factor κβ, conditioned place preference, swim

## Abstract

A high percentage of relapse to compulsive cocaine-taking and cocaine-seeking behaviors following abstinence constitutes a major obstacle to the clinical treatment of cocaine addiction. Thus, there is a substantial need to develop effective pharmacotherapies for the prevention of cocaine relapse. The reinstatement paradigm is known as the most commonly used animal model to study relapse in abstinent human addicts. The primary aim of this study is to investigate the potential effects of systemic administration of glucagon-like peptide-1 receptor agonist (GLP-1RA) exendin-4 (Ex4) on the cocaine- and stress-triggered reinstatement of cocaine-induced conditioned place preference (CPP) in male C57BL/6J mice. The biased CPP paradigm was induced by alternating administration of saline and cocaine (20 mg/kg), followed by extinction training and then reinstatement by either a cocaine prime (10 mg/kg) or exposure to swimming on the reinstatement test day. To examine the effects of Ex4 on the reinstatement, Ex4 was systemically administered 1 h after the daily extinction session. Additionally, we also explored the associated molecular basis of the behavioral effects of Ex4. The expression of nuclear factor κβ (NF-κβ) in the nucleus accumbens (NAc) was detected using Western blotting. As a result, all animals that were treated with cocaine during the conditioning period successfully acquired CPP, and their CPP response was extinguished after 8 extinction sessions. Furthermore, the animals that were exposed to cocaine or swimming on the reinstatement day showed a significant reinstatement of CPP. Interestingly, systemic pretreatment with Ex4 was sufficient to attenuate cocaine- and stress-primed reinstatement of cocaine-induced CPP. Additionally, the expression of NF-κβ, which was upregulated by cocaine, was normalized by Ex4 in the cocaine-experienced mice. Altogether, our study reveals the novel effect of Ex4 on the reinstatement of cocaine-induced CPP and suggests that GLP-1R agonists appear to be highly promising drugs in the treatment of cocaine use disorder.

## Introduction

Recent epidemiological data from the World Drug Report indicate that substance use disorder is spreading widely and rapidly, causing about 585,000 deaths worldwide per year (UNODC, 2019). Cocaine is considered one of the most commonly consumed illicit psychostimulants (Butler et al., 2017). Additionally, cocaine abuse is characterized by a high rate of relapse following a long period of detoxification. In abstinent human addicts, stressful life events and reexposure to the psychostimulants themselves are well known as two major stimuli for relapse to cocaine-associated behavior (Jaffe et al., 1989; O’Brien, 1997; Sinha et al., 1999). In fact, to date, the high relapse rate remains a major obstacle to the clinical treatment of cocaine-addicted individuals (O’Brien, 1997). Unfortunately, despite extensive efforts over the past decades to search for medications to treat cocaine addiction and reduce relapse, the effectiveness of available modulators has been reported to be limited. Thus, there is a substantial need for identifying more effective medications to treat cocaine use disorder and prevent relapse.

The glucagon-like peptide-1 (GLP-1) is traditionally thought to be a neuropeptide and circulating hormone found both in the nucleus tractus solitarius (NTS) neurons of the hindbrain (Alvarez et al., 1996) and in L cells of the intestines (Novak et al., 1987). While GLP-1 has a short duration of action, GLP-1 receptor (GLP-1R) agonists including exendin-4 (Ex4) display a longer half-life (Gallwitz, 2004) compared with GLP-1 and easily cross the blood-brain barrier (Holscher, 2010). Additionally, GLP-1R agonists can effectively regulate food intake (Hayes et al., 2008) as well as glucose-dependent insulin secretion (Holst and Seino, 2009), gastric emptying, and glucagon secretion (Matsuyama et al., 1988). Given these biological properties, Ex4 is clinically used for the treatment of type II diabetes mellitus and obesity (Andersen et al., 2018). Besides, Ex4 has also received increasing attention in recent years because of the suppressive actions on the maladaptive behavior in rodents including cocaine-induced CPP as well as locomotor activity and self-administration (Hernandez and Schmidt, 2019). However, few studies, thus far, have explored the potential effects of Ex4 on the cocaine- and stress-triggered reinstatement of cocaine-induced CPP and the expression of nuclear factor κβ (NF-κβ) in the nucleus accumbens (NAc).

Nuclear factor κβ is generally referred to as an important transcription factor due to its involvement in the transcription of many genes, such as those heavily implicated in immune responses and inflammation (Chen and Greene, 2004; Lin and Karin, 2007). These neuroinflammatory reactions are believed to play a pivotal role in neural adaptations after repeated consumption of psychostimulants (Kohno et al., 2019). Meanwhile, it is interesting to note that the anti-inflammatory agents appear highly promising in the treatment of substance use disorders (Kohno et al., 2019). For instance, previous studies indicated that inhibition of NF-κβ can block the rewarding effects of cocaine and the ability of previous cocaine exposure to augment the place preference of an animal for the cocaine-paired environment (Russo et al., 2009). Furthermore, the underlying benefits of some pharmacological agents including garcinol (Dunbar and Taylor, 2017; Monsey et al., 2017) and cannabidiol (de Carvalho and Takahashi, 2017) on cocaine-seeking behavior could also be attributed, at least partially, to an inhibition of neuroinflammation (e.g., direct and indirect inhibition of NF-κβ activation) and antioxidant (e.g., inhibition of iNOS) properties (Padhye et al., 2009; Kozela et al., 2017). Accordingly, modulation of NF-κβ signaling may be used as a promising strategy for addiction treatment. Importantly, other GLP-1R agonists have been confirmed to reduce the activation of the NF-κβ signaling by inhibiting the p65 subunit of the NF-κβ complex (Kozela et al., 2017; Ma et al., 2018). Therefore, it is conceivable that GLP-1R agonist Ex4 may attenuate cocaine- and stress-primed reinstatement of cocaine-mediated CPP and contribute to neuroplastic alternations in the NF-κβ-p65 expression in the cocaine-experienced mice.

In this study, we first evaluated the behavioral effects of the clinically available GLP-1R agonist Ex4 on the cocaine- and stress-triggered reinstatement of cocaine-induced conditioned place preference (CPP) in male C57BL/6J mice. In addition, the associated changes in the accumbal expression of NF-κβ-p65 were explored as well.

## Materials and Methods

### Animals

Male C57BL/6J mice (aged: 8–10 weeks, weighing 18–24 g) were obtained from the Experimental Animal Center of Ningxia Medical University (Yinchuan, China). They were maintained in specific pathogen-free animal cages on a reversed light cycle with lights on at 07:00 p.m. and lights off at 07:00 a.m. They were housed in groups (4 per cage) in a temperature-controlled room (23 ± 3°C), and standard rodent chow and tap water were available *ad libitum*. Behavioral testing was conducted during the light cycle of the mice. Mice were habituated for 1 week before any experimentation. To reduce the psychological stress provoked by an unfamiliar room, each mouse was habituated in the testing room for 30 min before the beginning of each behavioral testing. In this study, all experimental procedures involving animals and equipment were conducted according to the associated laws and regulations and were authorized by the Animal Research Ethics Committee of Ningxia Medical University.

### Reagents and Antibodies

The testing drug cocaine-hydrochloride (cocaine-HCl) was provided by China National Medicines Corporation Limited (Beijing, China). Cocaine-HCl was dissolved in 0.9% physiological saline solution (0.9% NaCl) to a concentration of 4 mg/ml and freshly prepared for daily injection. The volume of injection was 0.1 ml [(0.4 mg × 1 ml)/4 mg]. Saline was injected as a vehicle solution in this study. Ex4 was purchased from MedChemExpress (MCE, United States) and dissolved in 0.9% NaCl solution at a concentration of 0.04 μg/ml. It was administered at 2.0 μg/kg body weight and was prepared prior to daily injection. Ex4 and cocaine-HCl were alternatively administered on the left or right side of the peritoneum. Antibody for phospho-NF-κβ subunit p65 was purchased from Abcam (San Francisco, CA, United States). During the whole period of experiments, all antibodies and drugs were stored in refrigerators at −20 and 4°C, respectively.

### Conditioning Apparatus

The general procedure of this study was performed as depicted previously with slight modifications (Meye et al., 2016). In brief, the place-conditioning chambers consisted of two larger distinct compartments (24 cm × 14 cm × 30 cm) divided by a smaller intermediate compartment (7.0 cm × 7.0 cm × 30 cm) with two removable guillotine doors. On each test day, guillotine doors were raised, so the animals had access to move freely throughout the entire place-conditioning chambers during the habituation period and on the test days. However, the removable guillotine doors were closed during the conditioning and extinction training. One of the larger compartments was composed of four black walls and a smooth black floor, and the other compartment was composed of four white walls and a rough white floor covered with blue sandpaper. The smaller central compartment included two gray walls and a smooth floor leading into the corresponding compartment. Briefly, these compartments with different colors and floor textures provide animals with different visual and tactile cues when they receive cocaine or saline injections. Tracking of the mice in the apparatus was performed using an infrared video camera suspended about 1 m above the conditioning apparatus. The overhead infrared camera and computer were used to record the real-time positions of the mice and their movement throughout the three compartments. The time spent and total distance traveled in each chamber were recorded using a computerized video tracking system (behavior analysis software Smart 3.0; Panlab, Spain; supported by RWD Life Science Co. Ltd., China). The biased CPP protocol was used in this study.

### Preconditioning Stage (Days 1–3)

On days 1–2, the guillotine doors were raised, and all mice were placed in the smaller central compartment to freely move throughout the three compartments for 20 min prior to the commencement of the pre-test. On day 3, mice were placed in the middle area of the conditioning apparatus with the guillotine doors open, and their time spent in each compartment was recorded for 20 min to determine the baseline preference. As previously described in the literature, the white compartment (non-preferred compartment) was assigned as the cocaine-paired compartment, and the black one (preferred compartment) was assigned as the saline-paired compartment in our study (Meye et al., 2016). The CPP score was designed as a significant increase in the amount of time that mice spent in the cocaine-paired chamber after the conditioning or on the reinstatement test day compared with control mice (Sal–Sal group).

### Acquisition of Conditioned Place Preference (Days 4–12)

After completing the baseline preference, animals were randomly divided into two groups (saline group and drug group) for the CPP training. In detail, for the saline group (Group I), animals were given a daily injection of saline no matter which compartment the animals were placed in. For the treated group (Group II), mice were conditioned with saline or cocaine (20 mg/kg) for 20 min on alternate days. The place preference testing took place on day 12. On the drug-free test day, the removable guillotine doors were lifted, and mice were positioned in the center of the conditioning apparatus and allowed to freely explore for 20 min. After the mice completed the testing, they were returned to their cages. The CPP score is expressed as the time spent in the drug-paired chamber.

### Extinction of Conditioned Place Preference (Days 13–20)

Following the CPP test, animals were randomly separated into two groups (vehicle group and Ex4 group that was treated with Ex4 or saline 1 h after each extinction training, respectively), resulting in four independent groups: Sal + Sal, Coc + Sal, Sal + Ex4, and Coc + Ex4. During this time, half of the group of mice received daily administration of Ex4, and the other half received a daily injection of saline 1 h after they were confined in the previously designated cocaine-paired compartment for 20 min. Place preference for the cocaine-paired compartment was reexamined on day 20 to evaluate whether CPP was successfully extinguished after 8 extinction sessions. In this study, extinction criteria were considered to be achieved when there was no significant difference in the CPP scores in comparison with the Sal + Sal group.

### Reinstatement of Conditioned Place Preference (Day 21)

Following the extinction test, mice were reinstated by a swim protocol or a lower dose of cocaine (10 mg/kg, intraperitoneal [i.p.]). On the reinstatement test day, the guillotine doors were raised, and animals were provided free access to the entire conditioning apparatus for 20 min. The aim of the reinstatement test was to evaluate the effects of Ex4 pretreatment during extinction on the reinstatement of the cocaine-induced CPP.

#### Experiment 1

**FIGURE 1:**
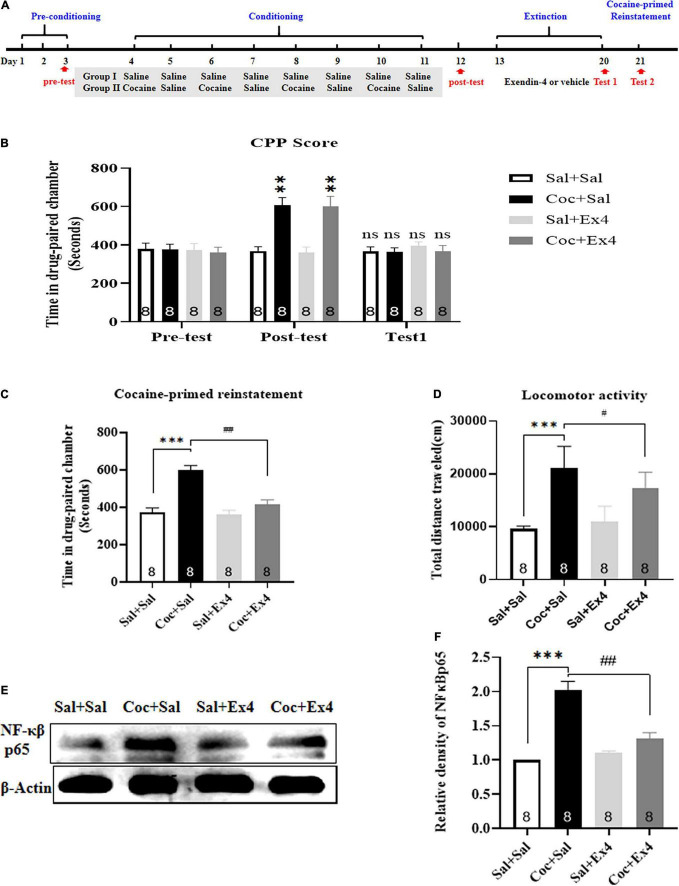
The systemic pretreatment with exendin-4 prevented cocaine-primed reinstatement of cocaine-induced CPP. **(A)** The experimental schedule for saline as well as cocaine and exendin-4 treatments. **(B)** After cocaine conditioning, animals displayed a significant place preference for the cocaine-paired compartment. ^**^ represents *p* < 0.01 vs Sal + Sal group, *n* = 8 each group, two-way rmANOVA. Additionally, CPP scores after vehicle or exendin-4 administration suggested that the cocaine-induced CPP was completely extinguished after 8 days extinction training. ns represents no significant, difference, *n* = 8 each group, two-way rmANOVA followed by Dunnett’s multiple comparisons test. **(C)** The systemic pretreatment with exendin-4 significantly prevented cocaine-primed reinstatement of cocaine-induced CPP. Half dose of cocaine on the reinstatement day induced a significant reinstatement CPP that was attenuated by The systemic pretreatment with exendin-4 after each extinction. ^***^ represents *p* < 0.001 vs Sal + Sal, ^##^ represent *p* < 0.01 vs Coc + Sal, *n* = 8 each group, two-way ANOVA followed by a Tukey’s *post hoc* test. **(D)** The systemic pretreatment with exendin-4 significantly attenuated locomotor activity. Half dose of cocaine on the reinstatement day induced a significant increase in locomotion that was attenuated by systemic pretreatment with exendin-4 after each extinction. ^***^ represents *p* < 0.001 vs Sal + Sal, ^#^ represent *p* < 0.05 vs Coc + Sal, *n* = 8 each group, two-way ANOVA followed by a Tukey’s *post hoc* test. **(E)** Representative image of NF-κβ p65 proteins in different groups detected by Western blot analysis at the end of CPP test of reinstatement (*n* = 8 in each group). **(F)** Semiquantitative analysis of the relative levels of NF-κβ p65 by densitometric analysis in different groups. Priming induced by cocaine up-regulated the expression of NF-κβ p65, and this effect was alleviated by systemic pretreatment with exendin-4 after each extinction. ^***^ represents *p* < 0.001 vs Sal + Sal, ^##^ represent *p* < 0.01 vs Coc + Sal, two-way ANOVA followed by a Tukey’s *post hoc* test. All data are presented as the mean ± SEM.

For the cocaine-primed reinstatement, mice in the saline-conditioned control group were primed with saline (2.5 ml/kg, i.p.), while animals in the cocaine-conditioned group were primed with a half dose of cocaine (10 mg/kg, i.p.). The time spent in the three compartments was recorded to assess the effects of Ex4 on the cocaine-primed reinstatement CPP. At the end of the CPP test of reinstatement, all mice were sacrificed using rapid decapitation for Western blotting. The NAc was extracted from the brain, snap-frozen in liquid nitrogen, and stored at −80°C until the initiation of Western blot. The timeline is shown in Figure 1A.

#### Experiment 2

**FIGURE 2:**
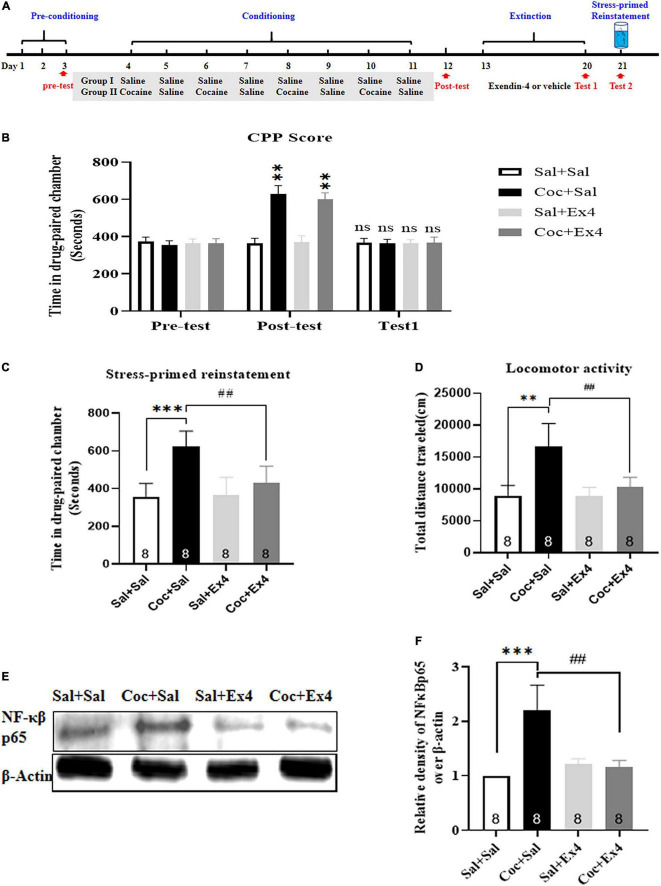
The systemic pretreatment with exendin-4 blocked stress-induced reinstatement of cocaine-induced CPP. **(A)** The experimental schedule for Swim, saline as well as cocaine and exendin-4 treatments. **(B)** After cocaine conditioning, animals developed a robust room preference for the cocaine-paired compartment. ^**^ represents *p* < 0.01 vs Sal + Sal group, *n* = 8 each group, two-way rmANOVA. Additionally, CPP scores after vehicle or exendin-4 administration suggested that the cocaine-induced CPP was completely extinguished after 8 days extinction training. ns represents no significant difference, *n* = 8 each group, two-way rmANOVA followed by Dunnett’s multiple comparisons test. **(C)** The systemic pretreatment of exendin-4 significantly blocked stress-induced reinstatement of cocaine-induced CPP. Swim on the reinstatement day induced a strong reinstatement CPP that was attenuated by systemic pretreatment with exendin-4 after each extinction. ^***^ represents *p* < 0.001 vs Sal + Sal, ^##^ represent *p* < 0.01 vs Coc + Sal, *n* = 8 each group, two-way ANOVA followed by a Tukey’s *post hoc* test. **(D)** The systemic pretreatment with exendin-4 significantly suppressed locomotor activity. Swim on the reinstatement day produced a clear increase in locomotion that was suppressed by systemic pretreatment with exendin-4 after each extinction. ^***^ represents *p* < 0.001 vs Sal + Sal, ^#^ represent *p* < 0.05 vs Coc + Sal, *n* = 8 each group, two-way ANOVA followed by a Tukey’s *post hoc* test. **(E)** Representative image of NF-κβ p65 proteins in different groups detected by Western blot analysis at the end of CPP test of reinstatement (*n* = 8 in each group). **(F)** Semiquantitative analysis of the relative levels of NF-κβ p65 by 1 densitometric analysis in different groups. Stress induced by Swim up-regulated the expression of NF-κβ p65, and this effect was alleviated by systemic pretreatment with exendin-4 after each extinction. ^***^ represents *p* < 0.001 vs Sal + Sal, ^##^ represent *p* < 0.01 vs Coc + Sal, two-way ANOVA followed by a Tukey’s *post hoc* test. All data are presented as the mean ± SEM.

The animal model of stress-induced reinstatement was established using a swim protocol as previously described in mice (Kreibich and Blendy, 2004; Carey et al., 2007; Redila and Chavkin, 2008; Mantsch et al., 2010; Meye et al., 2016). In brief, mice were required to swim in a 30 cm × 20 cm cylindrical polypropylene pool filled with water at a controlled temperature of 20–25°C for 6 min. The depth of the water was chosen based on the ability to touch the bottom of the pool with their hind limbs. Subsequently, mice were dried and recovered in their home cage for 20 min. The testing of stress-induced reinstatement was initiated by placing the animals in the middle area of the conditioning apparatus, where they were allowed to freely explore for 20 min. The time spent in the three compartments was recorded. At the end of the CPP test of reinstatement, the mice were sacrificed using rapid decapitation. The NAc tissue was removed from the brain, snap-frozen in liquid nitrogen, and stored at −80°C for Western blot. The timeline of experimental protocols is depicted in Figure 2A.

### Locomotor Activity

Locomotor activity is traditionally thought to be one of the most useful measurements to evaluate the behavioral effect of psychostimulants (Steketee and Kalivas, 2011). Locomotor activity, assessed by total distance, is automatically recorded to further evaluate the effects of systemic pretreatment with Ex4 on the cocaine-primed and stress-induced reinstatement when animals are subject to the 20-min reinstatement test in the conditioning compartments.

### Western Blotting Analysis

After completing all behavioral experiments, mice were sacrificed using rapid decapitation for Western blotting, and it was initiated to detect the expression level of NF-κβ-p65 proteins in the NAc. The NAc was chosen because it is known as a critical brain reward region implicated in cocaine reward (Hernandez et al., 2019). The general procedure of dissection of NAc tissue was conducted as previously described (Shin et al., 2003). Coronal sections of mouse brains were performed at prefrontal cortex (PFC) level and NAc/dorsal striatum level in a cooled 0.5 mm mouse brain matrix (Braintree, United States). Over ice glass, the PFC and dorsal striatum were carefully removed using forceps, whereas the NAc were dissected using a cooled 18-G micropunch, respectively. These NAc tissue samples were instantly frozen on dry ice and then stored at −80°C. The samples were homogenized on ice. Total protein was extracted using RIPA lysis buffer containing phenyl methane sulphonyl fluoride (PMSF), phosphatase inhibitors, and protease inhibitors (cat. no. KGP2100; KeyGEN Biotechnology Co., Ltd., Jiangsu, China). The protein sample was centrifuged for 5 min at 12,000 × *g* at 4°C. The protein concentration of the supernatants was measured using the BCA Protein Quantitation Assay Kit (KGPBCA; KeyGEN Biotechnology Co. Ltd., Jiangsu, China). Equal amounts of protein (50 μg per lane) from the Sal + Sal, Coc + Sal, Sal + Ex4, and Coc + Ex4 groups were separated using 10% sodium dodecyl sulfate-polyacrylamide gel electrophoresis (SDS-PAGE; cat. no. KGP113; KeyGEN Biotechnology Co. Ltd., Jiangsu, China) containing 30% acrylamide-methylene bisacrylamide, 1.0 mol/L Tris–HCl (*PH* = 6.8), 1.5 mol/L Tris–HCl (*PH* = 8.8), 10% ammonium persulfate, 10% SDS, TEMED, and then transferred to polyvinylidene difluoride membranes (Millipore, United States). After blocking with 5% skim milk powder for 2 h, the membranes were incubated in primary antibodies targeting NF-κβ-p65 (1:1,000; Abcam) and β-actin (1:2,000; Abcam) at 4°C overnight. After washing three times with TBST for 15 min, the membranes were incubated with the corresponding secondary antibody (1:1,000) for 2 h at room temperature. Immunoreactivity was visualized using the ECL Western blotting detection reagents and then analyzed through scanning densitometry. The proteins were detected using an enhanced chemiluminescence reagent, and the ratio of the gray value of the target protein band to the gray level of the β-actin band is used to quantify the relative expression level of the target protein.

### Statistical Analysis

All data are presented as means ± standard errors of the means. GraphPad Prism 8.4.0 software (GraphPad Software, La Jolla, CA, United States) was used to analyze all statistics in this study. The CPP score from the post-test and test 1 was analyzed using two-way repeated-measures analysis of variance (two-way rmANOVA) when compared with Sal + Sal group. The effects of systemic administration of Ex4 on the priming- and stress-induced reinstatement of cocaine-related CPP, locomotor activity, and the expression level of NF-κβ-p65 in the NAc were assessed using two-way analysis of variance (two-way ANOVA). *Post hoc* comparisons in the two-way rmANOVA were performed using Dunnett’s multiple comparisons test or Tukey’s multiple comparisons test. Differences with *p* values less than 0.05 were considered statistically significant.

## Results

### The Systemic Pretreatment With Exendin-4 During Extinction Blocked Cocaine-Primed Reinstatement of Cocaine-Induced Conditioned Place Preference and Nuclear Factor-κβ-p65 Expression in the Nucleus Accumbens

We first examined the effect of systemic pretreatment with Ex4 after each extinction session on the cocaine-primed reinstatement and NF-κβ-p65 expression in the NAc. A total of 35 mice were used in this experiment, with three mice excluded due to initial room bias or extinction training failure. As shown in Figure 1B, after completing 8 days of cocaine-mediated CPP training, there was a statistical significance in CPP scores among these groups [treatment: *F*_(3_,_28)_ = 6.061, *p* = 0.0027; test: *F*_(2_,_28)_ = 11.69, *p* = 0.0004; interaction *F*_(6_,_28)_ = 8.462, *p* < 0.0001; two-way rmANOVA], and Dunnett’s multiple comparison *post hoc* analysis showed that mice in the drug group that were treated with cocaine on alternating days displayed a significant increase in CPP scores compared with the Sal + Sal group (*p* = 0.0023 for Coc + Sal group, and *p* = 0.0085 for Coc + Ex4 group), indicating that cocaine-induced a robust preference for the cocaine-paired chamber and that a biased CPP paradigm was successfully established. Subsequently, animals from the saline and drug groups were randomly assigned to four independent groups: Sal + Sal, Coc + Sal, Sal + Ex4, and Coc + Ex4. All of them were required to undergo daily injection of Ex4 and extinction for 8 days. A two-way rmANOVA followed by Dunnett’s multiple comparison test showed that there was no significant difference in CPP scores compared with the Sal + Sal group (*p* > 0.05; *n* = 8 per group; Figure 1B), indicating that the cocaine-induced CPP response was completely extinguished. Afterward, these extinguished animals were reinstated with a half dose of cocaine (10 mg/kg) prior to the beginning of the reinstatement test. As expected, two-way ANOVA suggested a significant difference in the time spent in the cocaine-paired chamber [*F*_(3_,_28)_ = 21.82, *p* < 0.001; Figure 1C], and Tukey’s multiple comparison *post hoc* analysis showed that the Coc + Sal group developed a robust reinstatement of cocaine-induced CPP compared with the Sal + Sal group (*p* < 0.001) that was significantly attenuated by Ex4 (*p* < 0.001). Aside from CPP, two-way ANOVA also revealed a statistical difference in the locomotor activity [*F*_(3_,_28)_ = 33.56, *p* < 0.001; Figure 1D], and Tukey’s multiple comparison *post hoc* analysis showed that the Coc + Sal group presented a significant increase in locomotion compared with the Sal + Sal group (*p* < 0.001) that was significantly attenuated by Ex4 (*p* < 0.001). Overall, these findings showed that systemic pretreatment with Ex4 during extinction was sufficient to block cocaine-primed reinstatement of cocaine-induced CPP and hyperlocomotion.

At the end of the priming test, the NAc of animals was harvested to complete Western blotting. The Western blotting showed that the Sal + Sal, Coc + Sal, Sal + Ex4, and Coc + Ex4 groups had different relative protein contents of NF-κβ-p65 [*F*_(3_,_28)_ = 33.56, *p* < 0.001, two-way ANOVA; Figures 1E,F], and Tukey’s multiple comparison *post hoc* analysis showed that the Coc + Sal group had a significant increase in NF-κβ-p65 expression compared with that in the Sal + Sal group (*p* < 0.001), which was significantly reduced by Ex4 (*p* < 0.001). These results revealed that Ex4 normalized the abnormal expression of NF-κβ-p65 in the NAc.

### The Systemic Pretreatment With Exendin-4 During Extinction Inhibited Stress-Primed Reinstatement of Cocaine-Induced Conditioned Place Preference and Nuclear Factor-κβ-p65 Expression in the Nucleus Accumbens

We further explored the effect of systemic pretreatment with Ex4 after each extinction session on stress-induced reinstatement and NF-κβ-p65 expression in the NAc. A total of 37 mice were used in this experiment, with 3 and 2 mice excluded due to initial preference or extinction training failure, respectively. As shown in Figure 2B, following daily CPP training of cocaine for 8 days, there was a statistical significance in CPP scores among these groups [treatment: *F*_(3_,_28)_ = 6.931, *p* = 0.0006; test: *F*_(2_,_28)_ = 36.14, *p* < 0.001; interaction *F*_(6_,_28)_ = 7.716, *p* < 0.0001; two-way rmANOVA], and Dunnett’s multiple comparison *post hoc* analysis showed that animals in the drug group that were administered cocaine or saline on alternate days developed a significant increase in CPP score when compared with the Sal + Sal group (*p* = 0.0040 for Coc + Sal group, and *p* = 0.0028 for Coc + Ex4 group), indicating that the preclinical model of biased CPP was successfully induced. Next, we observed the effect of systemic pretreatment with Ex4 in the stress-induced reinstatement of CPP. Animals from the saline and drug groups were randomly split into four groups (Sal + Sal, Coc + Sal, Sal + Ex4, and Coc + Ex4) to experience daily administration of Ex4 and 8 extinction sessions. A two-way rmANOVA followed by Dunnett’s multiple comparison test showed that there was no significant difference in CPP scores compared with the Sal + Sal group (*p* > 0.05; *n* = 8 per group; Figure 2B), suggesting that the CPP induced by cocaine during conditioning period was fully extinguished. Subsequently, the extinguished animals were required to swim in a specific pool for 6 min and recover in their home cages for 20 min before the reinstatement test. As hypothesized, two-way ANOVA suggested a significant difference in the time spent in the cocaine-paired chamber [*F*_(3_,_28)_ = 14.77, *p* < 0.001; Figure 2C], and Tukey’s multiple comparison *post hoc* analysis showed that the Coc + Sal group developed a strong reinstatement of cocaine-induced CPP compared with the Sal + Sal group (*p* < 0.001) that was significantly attenuated by Ex4 (*p* = 0.0021), indicating that stress caused by swimming was able to produce a strong reinstatement CPP and this response was clearly reversed by systemic pretreatment with Ex4. Similar to the CPP responses, two-way ANOVA indicated an important difference in the locomotion [*F*_(3_,_28)_ = 26.82, *p* < 0.001; Figure 2D], and Tukey’s multiple comparison *post hoc* analysis suggested that the Coc + Sal group produced a significant increase in locomotion compared with the Sal + Sal group (*p* < 0.001) that was clearly reversed by Ex4 (*p* < 0.001). Altogether, these data showed that pretreatment with Ex4 during extinction was competent to inhibit stress-induced reinstatement of cocaine-induced CPP and locomotion.

Following the completion of the reinstatement test, these mice were sacrificed by cervical dislocation, and the NAc was collected for Western blotting. There was a different relative protein content of NF-κβ-p65 among the Sal + Sal, Coc + Sal, Sal + Ex4, and Coc + Ex4 groups [*F*_(3_,_28)_ = 41.08, *p* < 0.001, two-way ANOVA; Figures 2E,F], and then Tukey’s multiple comparison *post hoc* analysis suggested that the Coc + Sal group had a significant increase in NF-κβ-p65 expression compared with the Sal + Sal group (*p* = 0.0007) that was significantly diminished by Ex4 (*p* = 0.0015). These results revealed that Ex4 weakened the aberrant expression of NF-κβ-p65 in the NAc.

## Discussion

Reinstatement is the most commonly used measure to study the relapse in addicts (Vorel et al., 2001). Cocaine (Wit and Stewart, 1981) and stress (Erb et al., 1996) are identified as common relapse triggers. In this study, we, therefore, reveal the important effect of GLP-1R agonist Ex4 in the reinstatement evoked by cocaine and stress. Specifically, we demonstrate that systemic administration of Ex4 after each extinction training attenuates the cocaine- and stress-primed reinstatement of CPP. In addition, similar to reexposure to half dose of cocaine, we also observe that swimming following extinction induces a significant reinstatement of cocaine-mediated CPP. Moreover, animals experiencing cocaine- and stress-reinstatement show a significant increase in the accumbal expression of NF-κβ, which is also normalized by Ex4. Taken together, these findings suggest that GLP-1R agonist Ex4 has suppressing effects on the reinstatement of cocaine-induced CPP, and its pharmacological mechanism might be associated with the NF-κβ signaling inhibition.

### Glucagon-Like Peptide-1 Receptor Agonist Exendin-4 and Cocaine-Priming Reinstatement

One of the main findings of this study is the demonstration of the inhibitory actions of Ex4 on the reinstatement of cocaine-induced CPP. We show the biased CPP paradigm was successfully established using cocaine conditioning (20 mg/kg, i.p.), and then the CPP response is extinguished after the cocaine-free extinction sessions (8 days). Additionally, the significant reinstatement of cocaine-induced CPP is induced after reexposure to a half dose of cocaine (10 mg/kg, i.p.). These results are in line with our previous study showing that 20 and 10 mg/kg were sufficient to produce CPP and reinstatement, respectively (Zhu et al., 2021). Furthermore, this study also suggests that the systemic pretreatment with Ex4 after each extinction session significantly prevents the cocaine-priming reinstatement of the conditioned behavior in the study. These are in accordance with numerous findings indicating that these behavioral changes induced by cocaine consumption could be abolished *via* peripheral administration of Ex4 (Skibicka, 2013; Engel and Jerlhag, 2014; Hayes and Schmidt, 2016; Hernandez et al., 2019).

Besides, we also observe the possible effects of Ex4 on the locomotor activity, which is traditionally considered as another important paradigm used to measure the behavioral effects of psychostimulants (Steketee and Kalivas, 2011). In this study, reexposure to an injection of cocaine on the reinstatement test day markedly increases locomotion in mice, in accordance with previous evidence (Steketee and Kalivas, 2011). Importantly, systemic pretreatment with Ex4 induces a significant reduction in locomotion. Altogether, systemic administration of Ex4 after each extinction could exert a profound influence on the reinstatement of maladaptive behavior elicited by repeated cocaine use such as reinstatement CPP and locomotion. From this, our study indicates a crucial effect of GLP-1R agonist Ex4 in reducing reinstatement. However, less is known about the underlying impacts of Ex4 on the stress-induced reinstatement of cocaine CPP and the expression of plasticity-linked proteins including NF-κβ in the NAc.

### Glucagon-Like Peptide-1 Receptor Agonist Exendin-4 and Stress-Induced Reinstatement

Another major finding of our work is the evidence of inhibitory actions of Ex4 on the stress-induced reinstatement of cocaine CPP. In this experiment, animals also displayed a strong room preference that was extinguished after the 8 confined extinction sessions. In addition to reexposure to cocaine, numerous clinical studies showed that relapse to addictive behavior was often triggered by stressful life events (Khantzian, 1985; Shiffman and Wills, 1985; Kosten et al., 1986). Similarly, the stressful condition was commonly referred to as another important contributing factor of relapse to substance use disorder in abstinent addicts (Shaham et al., 2000). Furthermore, in preclinical studies, stress caused by swimming was also believed to enhance the rewarding effects of psychostimulant drugs (Piazza et al., 1990). In support of these studies, the CPP paradigm and swimming were widely used as stressors by previous studies (Vaughn et al., 2012; Tung et al., 2016). Accordingly, our data also suggested that exposure to swimming for 6 min prior to the commencement of the reinstatement test was sufficient to produce the significant reinstatement of CPP in the Coc + Sal group, which was congruent with a previous study (Kreibich and Blendy, 2004; Carey et al., 2007; Redila and Chavkin, 2008; Mantsch et al., 2010; Meye et al., 2016). Intriguingly, the stress-induced reinstatement of previously extinguished cocaine CPP was clearly attenuated by systemic pretreatment with Ex4 after each extinction training in the Coc + Ex4 group.

In addition, this study also demonstrates that exposure to swimming can markedly increase locomotion in animals, in accordance with a previous study (Meye et al., 2016). Similarly, Ex4 also induces a significant reduction in locomotor activity in the stress-induced reinstatement paradigm. From these, the stress-induced reinstatement does not only develop a significant CPP but also increases locomotion, and this response is attenuated by Ex4 pretreatment.

Taken together, the results of this work were provocative and revealed the inhibitory effect of GLP-1R agonist Ex4 in the two reinstatement paradigms of cocaine-induced CPP. However, the associated mechanisms of pharmacological actions of Ex4 on inhibiting stress-induced reinstatement are still unknown to date.

### Neuroinflammation and Reinstatement

In recent years, neuroinflammation has received considerable attraction due to its implication in many brain diseases (Block and Hong, 2005; Tansey et al., 2007). Furthermore, exposure to stressors contributes to a series of changes in neuroimmune response (Frank et al., 2019). In addition, cocaine is reported to enhance the activation of NF-κβ signaling in the reward area of the brain including NAc, and the signaling pathway can exert profound impacts on controlling the morphology of NAc neurons as well as attenuating the rewarding effects of cocaine (Russo et al., 2009). NF-κβ is an important transcription factor due to its involvement in inflammation and immune responses (Chen and Greene, 2004; Lin and Karin, 2007). Importantly, the anti-inflammatory agents are reported to act as a novel and effective approach for behavioral treatment of substance use disorders (Kohno et al., 2019). More interestingly, the pharmacological mechanism by which many inhibitors of neuroinflammation significantly attenuate the rewarding effects of cocaine are believed to be closely associated with direct and indirect inhibition of NF-κβ activation (Padhye et al., 2009; Kozela et al., 2017). Considering the significance of NF-κβ signaling in cocaine reward (Russo et al., 2009), we examined the effects of Ex4 on the expression of NF-κβ in cocaine-priming or stress-induced reinstatement animals. Our data show that cocaine exposure is adequate to upregulate NF-κβ signaling that may be normalized by systemic pretreatment with Ex4, in agreement with previous studies suggesting that another GLP-1R agonist liraglutide effectively inhibited the NF-κβ signaling pathways (Liu et al., 2008; Kozela et al., 2017; Ma et al., 2018; Li et al., 2020). Consequently, the behavioral effects of Ex4 on cocaine might be closely associated with the NF-κβ signaling inhibition.

Of note, there is a range of limitations and questions in this study. For example, many neurotransmitters such as dopamine, norepinephrine, and serotonin reportedly had a far-reaching effect on the rewarding and reinforcing properties of cocaine (Ritz et al., 1990). Except for the NAc, a growing body of studies also revealed that systemic administration of Ex4 was also expressed in other brain regions of the reward system such as the ventral tegmental area (VTA) and lateral septum (LS) (Hernandez and Schmidt, 2019). Accordingly, future experimental studies are urgently needed to validate the specific mechanisms underlying cocaine-primed and stress-induced reinstatement of cocaine CPP. Furthermore, to date, the precise mechanisms by which Ex4 exerted suppressive effects on cocaine- and stress-primed reinstatement remain unknown although the CPP score and expression of NF-κβ were decreased in the Coc + Ex4 group. One possible explanation is that the expression of NF-κβ was already enhanced by repeated exposure to cocaine prior to the reinstatement test due to previous studies showing that cocaine was shown to effectively induce an increase in the expression of NF-κβ in the NAc (Russo et al., 2009). And the upregulated expression of NF-κβ was reduced by Ex4 treatment, but this effect may have occurred before the beginning of the reinstatement test. Consequently, other possible factors such as the reinstatement test, drugs, and CPP should be included in future research to precisely elucidate the pharmacological mechanism underlying the behavioral effects of Ex4 on the cocaine- and stress-induced reinstatement. Moreover, evaluating the ability of Ex4 to affect the reinstatement of cocaine CPP by modifying the activity of specific neuromodulators in areas of the brain reward system is another problem that must be addressed. Additionally, it is not fully understood whether NF-κβ signaling in the NAc is an independent mechanism through which the Ex4 regulates the reinstatement of cocaine CPP as only four groups (Sal + Sal, Sal + Ex4, Coc + Sal, and Coc + Ex4) are included in this study. Furthermore, experimental studies are necessary to evaluate other pharmacological mechanisms of action of Ex4 in the brain for the treatment of cocaine use disorder. Besides, the important factor of sex differences in the efficacy of Ex4 in reducing the rewarding and reinforcing effects of cocaine is fully ignored as only the adult male mice are used in this study. Additional researches are essential to evaluate whether other GLP-1R agonists have a similar potential as Ex4 on other psychostimulant abuse. Finally, although the changes in NF-κβ expression after using Ex4 could be clearly observed in this study, the corresponding changes should be further investigated in human clinical experiments.

In summary, this study shows that reexposure to cocaine and stress induced by swimming on the reinstatement day markedly augmented the time spent in the cocaine-paired chamber, increased locomotor activity, and facilitated the expression of NF-κβ in the NAc. Importantly, these alterations could be prevented by systemic pretreatment with Ex4 after each extinction session. Our findings provide innovative insights into the therapeutic potential of the clinically available Ex4 for the treatment of cocaine relapse. Therefore, GLP-1R agonists including Ex4 may be a promising approach to treat substance use disorder.

## Data Availability Statement

The original contributions presented in the study are included in the article/Supplementary Material, further inquiries can be directed to the corresponding authors.

## Ethics Statement

The animal study was reviewed and approved by Animal Research Ethics Committee of Ningxia Medical University.

## Author Contributions

HG, TS, and FW participated in designing the experiments, preparation of the manuscript, analysis of data, and in the revision. CZ designed and performed the experiments, analyzed data, made the figures, performed the statistical analysis, and drafted the manuscript. All authors participated in the study design, data collection, analysis of data, and preparation of the manuscript, took responsibility for the integrity of the data and the accuracy of the data analysis, contributed to the article, read, and approved the final manuscript and approved the submitted version.

## Conflict of Interest

The authors declare that the research was conducted in the absence of any commercial or financial relationships that could be construed as a potential conflict of interest.

## Publisher’s Note

All claims expressed in this article are solely those of the authors and do not necessarily represent those of their affiliated organizations, or those of the publisher, the editors and the reviewers. Any product that may be evaluated in this article, or claim that may be made by its manufacturer, is not guaranteed or endorsed by the publisher.
